# Enhancing Direction-of-Arrival Estimation with Multi-Task Learning

**DOI:** 10.3390/s24227390

**Published:** 2024-11-20

**Authors:** Simone Bianco, Luigi Celona, Paolo Crotti, Paolo Napoletano, Giovanni Petraglia, Pietro Vinetti

**Affiliations:** 1 Department of Informatics, Systems and Communication, University of Milano-Bicocca, 20126 Milan, Italy; simone.bianco@unimib.it (S.B.); p.crotti1@campus.unimib.it (P.C.); paolo.napoletano@unimib.it (P.N.); 2 MBDA Missile Systems, 80070 Fusaro, Italy; giovanni.petraglia@mbda.it (G.P.); pietro.vinetti@mbda.it (P.V.)

**Keywords:** direction-of-arrival (DOA) estimation, convolutional neural networks, multi-task learning, ordinal regression

## Abstract

There are numerous methods in the literature for Direction-of-Arrival (DOA) estimation, including both classical and machine learning-based approaches that jointly estimate the Number of Sources (NOS) and DOA. However, most of these methods do not fully leverage the potential synergies between these two tasks, which could yield valuable shared information. To address this limitation, in this article, we present a multi-task Convolutional Neural Network (CNN) capable of simultaneously estimating both the NOS and the DOA of the signal. Through experiments on simulated data, we demonstrate that our proposed model surpasses the performance of state-of-the-art methods, especially in challenging environments characterized by high noise levels and dynamic conditions.

## 1. Introduction

Direction-of-Arrival (DOA) estimation is a task that finds applications in various fields, including acoustics, wireless communications, sonar, and radar [1,2,3,4]. To address this problem, several methods have been proposed that can be grouped into traditional methods [1,5,6] and learning-based methods, which are mainly based on deep learning models [7,8]. DL methods have shown impressive advantages when the amount of data used for training is sufficiently large and, most importantly, if the data used for training are representative of the reference scenario [9]. From a strictly technical point of view, DOA estimation is a regression problem where, in the most general case, the dimension of the target is not known a priori. This makes it challenging to apply traditional machine learning methods, which typically require training a model for each source. This issue can be encountered in approaches that use models such as Support Vector Regressors (SVRs) [10] or Support Vector Machine (SVM) [11]. There are also strategies that employ Radial Basis Functions (RBFs) [12,13]. In the latter case, the Number of Sources (NOS) is fixed and assumed to be known a priori, while in the former, which considers the 2D scenario, the problem is addressed by dividing the DOA grid into spatial sub-regions.

Previous methods have relatively good performance in situations where the noise power is very low, but the quality of the estimates degrades as the Signal-to-Noise Ratio (SNR) decreases and approaches zero (i.e., when the noise power is similar to that of the signal). Recently, several solutions have been proposed that utilize Deep Neural Networks (DNNs) [14]. These solutions employ various types of architectures, including Fully Connected Neural Networks (FCNNs) [9,15,16], Convolutional Neural Networks (CNNs) [17,18,19,20,21], and Recurrent Neural Networks (RNNs) [8,22]. The different approaches not only differ in the type of architecture, but also in the input used. Some solutions employ the covariance matrix (separating real and imaginary parts), sometimes vectorized [23,24], or distributed over multiple channels using CNNs [18,25], while others utilize complex-valued spatiotemporal neural networks [7]. Conversely, some methods directly use the received signal or operate in the frequency domain [26,27]. Several methods combine the source detection phase with the DOA determination phase or propose separate solutions for performing the two tasks. Some of these methods assume the number of signals to be known a priori [15,25]. In this regard, some neural models have been presented as replacements for traditional model order estimation methods for identifying the NOS [28,29]. In [28], two distinct types of input are evaluated: the real-valued vector consisting of the real and imaginary parts of the signal, and the covariance matrix of the received signals. In [29], the eigenvalues of the received signal’s covariance matrix are used as input for a regression network (ERNet) and for a classification network (ECNet). A similar strategy can be found in [26], where a neural regression model is implemented with multiple output branches, one of which is specialized in identifying the NOS. The adoption of deep learning-based approaches enhances performance and provides scalable, adaptable solutions that seamlessly integrate with other DOA-related tasks like AESA beamforming [30].

In this paper, we present a novel CNN architecture designed to receive the covariance matrix of the source waveform as input and perform simultaneous estimation of the NOS and DOA. Our approach treats DOA estimation as a regression problem, while NOS estimation leverages an ordinal regression technique. The use of ordinal regression overcomes the limitation of previous methods by considering the relative distance between an erroneously predicted data sample and its ground-truth label. The model is trained end-to-end in a multi-task manner, enhancing its ability to utilize shared representations. This is especially useful in complex settings where traditional methods falter due to variable signal patterns, as it allows the model to understand and integrate the interactions and dependencies between the NOS and DOA tasks. To thoroughly evaluate the robustness and effectiveness of our proposed method, we conduct a comprehensive series of experiments. These tests assess the model’s performance on simulated data with characteristics different from those in the training dataset.

The rest of this paper is as follows. A review of DOA methods is given in Section 2. Section 3 describes the signal model. Section 4 details the proposed multi-task CNN for DOA estimation. Section 5 and Section 6 present simulation results and conclusions, respectively.

## 2. Related Work

The beamforming method, a fundamental technique for DOA estimation, extends the Discrete Fourier Transform (DFT) from the time domain to the spatial domain. However, it becomes impractical when the beamwidth exceeds the spacing between the sources [1]. Following the development of beamforming, various methods based on spatial spectral estimation and maximum likelihood [31] were introduced for DOA estimation. Notable examples include the MUltiple SIgnal Classification (MUSIC) algorithm [5], the Estimation of Signal Parameters via Rotational Invariance Techniques (ESPRIT) algorithm [32], and their variations. The MUSIC algorithm is widely used for DOA estimation, constructing spatial spectral peaks by separating the signal and noise subspaces, which are orthogonal. The DOA can then be estimated from the corresponding spectral peaks using this orthogonal property. The CS-MUSIC algorithm [33], a variant of MUSIC, combines hybrid parametric minimization with MUSIC to address coherence limitations in DOA estimation. ESPRIT, another popular method, splits the array into two identical subarrays with corresponding elements paired at equal distances. This structure ensures that the incident angle differs by only one rotationally invariant factor between the two subarrays, allowing the DOA to be determined by solving for a generalized eigenvalue. Unitary ESPRIT [34], a variant of ESPRIT, exploits the centrosymmetric property of isometric linear matrices, transforming complex covariance matrices into real symmetric ones. This reduces computational complexity and enhances accuracy in DOA estimation. Extended-Phase Interferometry (EPI) combines phase and amplitude data to improve robustness in noisy environments. Using extended least-squares, EPI enhances accuracy over traditional phase interferometry while keeping computational demands moderate [35]. In [36], a low-power AoA estimation system has been proposed for IoT networks like LoRa. Phase data are sent to the cloud for processing, enabling a lightweight, cost-effective design suited for power-constrained applications with limited local processing. A fully digital, reconfigurable FPGA-based architecture for AoA estimation using phase interferometry, focused on low-latency real-time processing, has been proposed in [37]. Its modular design minimizes computational delays, making it ideal for applications needing rapid, precise updates, especially in environments benefiting from hardware-based signal processing.

Another class of DOA estimation techniques relies on sparse representation. In [6], a sparse representation-based method was proposed, using sensor measurements to promote sparsity and improve estimation accuracy via the $l_{1}$-norm penalty. In [38], compressive sensing was applied to DOA estimation, where the method estimates DOA by reconstructing the sparse signal vector [39]. A gridless DOA estimation approach based on a covariance fitting criterion was introduced in [40], offering continuous parameter estimation over time. This method avoids the need for partitioning the angular space into small grids, thus eliminating discretization issues.

In recent years, deep learning [41] has been applied to radio signal processing tasks, including signal detection [42,43], modulation recognition [44,45,46], channel estimation [47], and information recovery [48]. Similarly, deep learning-based methods have emerged as promising alternatives to traditional Direction-of-Arrival (DOA) estimation techniques [24,49]. Many of these methods approach the problem through classification models [17,18,24,49,50,51]. In [24], the authors tackled DOA estimation using a Deep Neural Network (DNN) with the lower triangular part of the covariance matrix as input for two signal sources. The network was trained in parallel on two different datasets: one with randomly generated signal angles and another with signal angles restricted to 1° intervals. A prediction was considered successful if at least one of the two DNNs accurately estimated the DOAs. However, no standardized procedure for selecting the appropriate DNN architecture for this problem was proposed. In [49], another DNN was used for estimating the DOAs of two sources, with four intermediate layers and nine possible choices for the number of units in each. A total of 36 DNNs were trained, and the one yielding the highest accuracy was chosen for DOA estimation. In [17], a deep convolutional network was employed to estimate DOA under sparse prior conditions. This approach reconstructed the spatial spectrum by analyzing the covariance matrix data, and a Convolutional Neural Network (CNN) was trained to extract spectral features. Different activation functions were tested in the hidden layers to optimize performance, while spatial sparsity was leveraged to improve accuracy compared to earlier methods. The design and training of the network were further enhanced by integrating prior information. The authors of [50] introduced CNN-based DeepMUSIC, a deep learning framework that divides the DOA region into sub-regions, with each sub-region handled by a specialized CNN for more precise DOA estimation. In [21], DOA is formulated as a multi-label classification problem, where the angular space is discretized into a grid of multiple classes, and a CNN is used to estimate the angles of arrival of the incoming source signals. In [18], the challenge of DOA estimation in low-Signal-to-Noise Ratio (SNR) conditions was addressed by using multichannel data as input, representing the real part, imaginary part, and phase of the covariance matrix as three separate channels. A 2D convolutional layer was used to extract meaningful features for more accurate DOA estimation. In [51], an online DOA estimation method was proposed by combining CNN with Long Short-Term Memory (LSTM) networks. This hybrid approach enhanced DOA estimation by capturing temporal dependencies in the data.

Deep learning-based DOA estimation methods can also be designed by combining encoders with neural networks. In [9], a method was introduced using a combination of self-encoder and Fully Connected Neural Networks, specifically a Multilayer Perceptron (MLP). This approach involved using a multilayer self-encoder to divide the signal into spatial sub-regions, similar to a spatial filter, and employing a classifier for spatial spectrum estimation in each sub-region. The DNN model was trained with array-specific outputs, making it robust against array imperfections. In [52], a model based on residual networks (ResNet) was proposed to address array defects. The model detected signals within sub-regions through a spatial classification network, followed by classification of outputs from different sub-regions using ResNet, leading to improved accuracy in DOA estimation.

Finally, in [53], a compressed data-based neural network model was introduced, which used joint training of an encoder and classifier for accurate DOA estimation. This model took encoded data from each subarray as input to the classifier, enabling accurate estimation even with limited data. To further simplify the process and avoid computing the covariance matrix, Zheng et al. [54] proposed directly using the raw in-phase and quadrature components of the signal as input to a DNN for DOA estimation.

## 3. Signal Model

**Figure 1:**
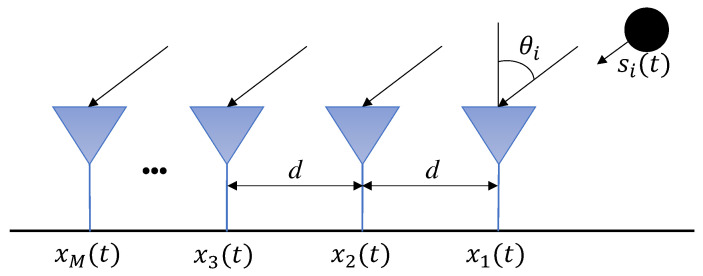
Uniform Linear Array (ULA); *d* is the distance between the sensors; $\theta_{i}$ is the angle of arrival of the impinging signal, and *M* is the number of sensor array antennas.

Consider a Uniform Linear Array (ULA) composed of *M* physical sensors spaced at a distance *d* from each other [55] as shown in Figure 1.

Assuming a scenario where there are *K* coplanar far-field narrowband signals, $s\left(t\right)=\left[s_{1},s_{2},\ldots ,s_{K}\right]$, coming from distinct directions $\theta =\left[\theta_{1},\theta_{2},\ldots ,\theta_{K}\right]$ impinging on the array. The signal received by the array at snapshot *t* is expressed as

(1) $$x_{\Omega }\left(t\right)=A_{\Omega }\left(\theta \right)s\left(t\right)+n_{\Omega }\left(t\right),\;t=1,2,\ldots ,T,$$

where $\Omega =\{\Omega_{1},\Omega_{2},\ldots ,\Omega_{M}\}$ denotes the index set of array sensors with $\Omega_{m}$ being the position of the *m*-th sensor with ordered rank $\Omega_{1}<\Omega_{2}<\ldots <\Omega_{M}$. $s\left(t\right)\in R^{K\times 1}$ and $n_{\Omega }\left(t\right)\in R^{M\times 1}$ denote the transmitted signal and the additive Gaussian sensor noise at the snapshot *t*, respectively. Let $A_{\Omega }\left(\theta \right)=\left[a_{\Omega }\left(\theta_{1}\right),a_{\Omega }\left(\theta_{2}\right),\ldots ,a_{\Omega }\left(\theta_{K}\right)\right]$ be the M×K manifold matrix of the signal direction vectors with

(2) $$a_{\Omega }\left(\theta \right)={\left[1,e^{j\frac{2\pi d}{\lambda }\sin\theta },e^{j2\frac{2\pi d}{\lambda }\sin\theta },\ldots ,e^{j\left(M-1\right)\frac{2\pi d}{\lambda }\sin\theta }\right]}^{⊤},$$

being the M×1 steering vector, where $j=\sqrt{-1}$, λ is the wavelength, and *d* is the array interval. Therefore, the covariance matrix of the received signal $x_{\Omega }\left(t\right)$ is

(3) $$R_{\Omega }=E\left[x_{\Omega }\left(t\right)x_{\Omega }^{H}\left(t\right)\right]=A_{\Omega }\left(\theta \right)R_{ss}A_{\Omega }^{H}\left(\theta \right)+R_{nn},$$

where E(·) and ${\left(\cdot \right)}^{H}$, respectively, denote the expectation operator and the Hermitian transpose (conjugate transpose) of a matrix. $R_{ss}=E\left[s\left(t\right)s^{H}\left(t\right)\right]$ denotes the covariance matrix of the source waveform, and $R_{nn}=\sigma^{2}I_{M}$ represents the noise covariance matrix with $\sigma^{2}$ the noise power and $I_{M}$ the M×M identity matrix.

## 4. The Proposed Method

**Figure 2:**
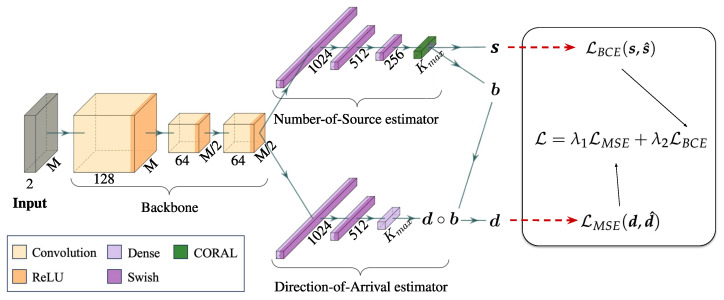
Architecture of the proposed multi-task CNN for DOA estimation. The network processes the signal covariance matrix through the backbone. The resulting feature vector is passed to two branches: the Number-of-Source estimator predicts the Number of Sources b (i.e., a binarized version of the logits s); the Direction-of-Arrival estimator provides multiple angles of arrival, denoted as d, corresponding to the number of angles b predicted by the other branch. A compound loss L is used to optimize the model based on the two task-specific losses.

The proposed model is depicted in Figure 2. It takes a three-dimensional matrix as input, where the spatial dimensions correspond to the covariance matrix, while the two channels represent the real and imaginary parts of the matrix. The model architecture comprises three convolutional blocks, which are shared between two branches, each specialized in distinct tasks. One branch is responsible for identifying the NOS, while the second branch handles the DOA estimation.

In our method, DOA estimation is formulated as a regression problem, while source counting is achieved by using ordinal regression. Specifically, we exploit the COnsistent RAnk Logits (CORAL) framework [56]. The ground-truth NOS $\hat{K}=\left|y\right|$ is first extended into $K_{max}-1$ binary labels ${\hat{s}}^{\left(1\right)},\ldots ,{\hat{s}}^{\left(K_{max}-1\right)}$ such that ${\hat{s}}^{\left(k\right)}\in \left\{0,1\right\}$ indicates whether ${\hat{s}}^{\left(k\right)}$ exceeds rank $r_{k}$. The predicted rank index is given by

(4) $$K=1+\sum_{k=1}^{K_{max}-1}b_{k},$$

where $b_{k}\in \left\{0,1\right\}$ is the prediction of the *k*-th binary classifier in the output layer. The required rank-monotonicity is achieved by using a dense layer that maps features into a single value and $K_{max}-1$ bias parameters.

The CORAL framework suits this application by treating NOS estimation as a ranked prediction problem, where the model distinguishes ordinal categories of source counts. This approach leverages ordinal relationships, capturing the incremental nature of source count estimation and reducing prediction error by considering the distance between predicted and actual counts, enhancing NOS estimation even in challenging cases.

### 4.1. Preprocessing

The covariance matrix $R_{\Omega }$ is unknown and is usually replaced by its sampled estimate ${\hat{R}}_{\Omega }$ which can be calculated according to the *T* snapshots. The input data ${\bar{R}}_{\Omega }$ of our model are real-valued M×M×2 tensors, where the third dimension represents different channels. In particular, the first channel is the real part of ${\hat{R}}_{\Omega }$:

(5) $${\bar{R}}_{\Omega }\left[:,:,1\right]=Re\left\{{\hat{R}}_{\Omega }\right\};$$

the second channel is the imaginary part of ${\hat{R}}_{\Omega }$:

(6) $${\bar{R}}_{\Omega }\left[:,:,2\right]=Im\left\{{\hat{R}}_{\Omega }\right\}.$$

Compared to the input of the model in [18], we normalize the input by using a $1/\left|\right|{\bar{R}}_{\Omega }{\left|\right|}_{2}$. This choice is motivated by the fact that, in this way, the model is more resilient to variations in the input’s order of magnitude.

### 4.2. Architecture

In this section the architecture of the proposed method for DOA estimation is presented. First, the backbone architecture is detailed, then a description of the Number-of-Source estimator and the Direction-of-Arrival estimator is given.

#### 4.2.1. Backbone

The backbone of the proposed model is a shared CNN between the two tasks, which is formalized as follows:

(7) $$\begin{array}{c} f_{B}\left({\bar{R}}_{\Omega }\right)=f_{3}\left(f_{2}\left(f_{1}\left({\bar{R}}_{\Omega }\right)\right)\right)=e. \end{array}$$

Each function ${\left\{f_{i}\left(\cdot \right)\right\}}_{i=1,2,3}$ represents a series of layers: a 2D convolutional layer with 128, 64, and 64 filters, respectively, followed by batch normalization, and a ReLU activation. The output of the last layer of the backbone, e, is flattened and passed to subsequent layers for further processing. A detail of each processing step of the backbone is given in Table 1.

#### 4.2.2. Number-of-Source Estimator

The branch that performs ordinal regression to identify the NOS takes as input the feature vector calculated by the backbone and estimates the NOS, $n=h_{NoS}\left(e\right)$. The NOS branch comprises four blocks. The first three blocks consist of a dense layer with 1024, 512, and 256 channels, respectively, followed by a Swish activation function [57]. The branch ends with the CORAL rank predictor layer. This last layer outputs a vector $s=\{s_{1},s_{i},\ldots ,s_{K_{max}}\}$ with $s_{i}\in R$. The NOS *K* is obtained by applying Equation (4). The binary predictions, $b=\{b_{1},b_{i},\ldots ,b_{K_{max}}\}$, are obtained via $b_{i}=1\left\{s_{i}\geq 0.5\right\}$.

#### 4.2.3. Direction-of-Arrival Estimator

The branch that estimates the DOA consists of two dense blocks, similar to those in the ordinal branch but with dropout layers (dropout rate of 0.2), followed by an output dense layer. More in detail, the two dense layers have 1024 and 512 channels, respectively. The last dense layer maps the 512-dimensional feature vector into $K_{max}$ real values to form the vector $d=\{d_{1},d_{i},\ldots ,d_{K_{max}}\}$ with $d_{i}\in R$ of the angles of arrival. The final DOA prediction is derived through element-wise multiplication between the binary vector of the NOS, b, and the vector containing the arrival angles, d, as follows d=d∘b.

### 4.3. Training Procedure

The proposed model is trained end-to-end for 200 epochs with a batch size of 32 samples by using the Adam optimizer [58], with an initial learning rate of 0.001, which is reduced by 0.5 every ten epochs. The early stopping technique is implemented to terminate training if the Root Mean Squared Error (RMSE), evaluated on the validation dataset, shows no improvement for 20 consecutive epochs.

Our model is optimized by exploiting the following loss function:

(8) $$\begin{array}{c} L=\lambda_{1}L_{MSE}+\lambda_{2}L_{BCE}, \end{array}$$

where $\lambda_{1}$ and $\lambda_{2}$ are empirically set to 1.0 and 0.8, respectively. It consists of the Mean Squared Error (MSE) calculated only on the angles predicted for the first *K* signals identified by the NOS branch as follows:

(9) $$\begin{array}{c} L_{MSE}\left(d,\hat{d}\right)=\frac{1}{NK}\sum_{n=1}^{N}\sum_{k=1}^{K}{\left({\hat{d}}_{k}^{\left(n\right)}-d_{k}^{\left(n\right)}\right)}^{2}, \end{array}$$

where *N* is the number of samples, *K* is the number of identified signals, and d and $\hat{d}$ are the predicted and the ground-truth angles, respectively. The second loss of Equation (8) is the Binary Cross-Entropy (BCE), which is defined as follows:

(10) $$\begin{array}{c} L_{BCE}\left(s,\hat{s}\right)=\\ -\frac{1}{NK}\sum_{n=1}^{N}\sum_{k=1}^{K_{max}-1}\log\left(s_{k}^{\left(n\right)}\right){\hat{s}}_{k}^{\left(n\right)}+\log\left(1-s_{k}^{\left(n\right)}\right)\left(1-{\hat{s}}_{k}^{\left(n\right)}\right), \end{array}$$

where *N* is the number of samples, $K_{max}$ is the maximum number of signals, *s* and $\hat{s}$ are the predicted and the ground-truth logits, respectively.

## 5. Simulations and Analysis of the Results

### 5.1. Simulation Settings

The samples used for training and evaluating the models are generated following the data model presented in Section 3.

For the generation of a dataset sample, the number of signals *K* is first randomly generated from $\left\{1,\ldots ,K_{max}\right\}$, then *K* angles are randomly sampled from $\left[-\theta^{\circ },+\theta^{\circ }\right]$. The covariance matrix $R_{\Omega }$ can be then estimated by exploiting the previous values and the fixed parameters of the ULA. The label associated with the covariance matrix is $y=\left[\theta_{1},\ldots ,\theta_{K}\right]$ Hence, each sample consists of a pair $\left(R_{\Omega },y\right)$. The parameters used in our simulations are designed to closely reflect real-world scenarios, incorporating appropriate approximations as recommended in [59]. Specifically, the ULA in this work consists of 10 elements (M=10) with an inter-element spacing of d=0.15, corresponding to a wavelength of λ=0.3. Additionally, we set the maximum number of impinging signals to $K_{max}=3$. We assume a sampling rate twice the signal frequency, following the Nyquist criterion to prevent aliasing [60]. We use a 32-bit ADC resolution. Finally, multipath components were set to zero, focusing on direct line-of-sight signals to isolate DOA estimation performance under controlled conditions.

We generate a dataset containing data samples with angles in the range $\left[-60^{\circ },+59^{\circ }\right]$, affected by variable SNRs uniformly sampled from the intervals [−13,−7], [−3,+3], and [+7,+13] dBs. Each sample consists of a total of T=2000 snapshots.

The generation of data samples begins by generating the entire DOA grid in the range of $\left[-60^{\circ },+59^{\circ }\right]$ with a step size of 1°. From this grid, all possible combinations of 1, 2, and 3 sources are created. For combinations involving two or three sources, the DOA sequences are randomly permuted. This process yields a total of 288,100 DOA combinations. For each DOA combination, three samples are generated for each one of the three SNR intervals. Considering the DOA grid, the various SNRs and the three sample variants, the dataset comprises 2,592,900 samples. We randomly split this dataset into training and validation sets, allocating 80% for training and 20% for validation. Consequently, the training set consists of 2,074,320 samples, while the validation set contains 518,580 samples.

We then generate seven distinct test sets to assess the method’s performance on unseen data and its capability to generalize to scenarios beyond those simulated in the training set. These test sets incorporate varying conditions—such as phase shifts, modulation types, and carrier frequencies—that impact both the signal waveform and the resulting covariance matrix. Since the covariance matrix captures the correlation structure of signals received across array sensors, these conditions affect the input to the proposed model in unique ways, altering spatial and temporal correlations.

T1. Same characteristics as the training set

This test set is designed with the same characteristics as the training set.

T2. Different number of snapshots

This test set contains samples with the same characteristics as the training set in terms of grid of arrival angles and noise levels but varies the number of snapshots *T* with values {5,100,200,500,1000}. Testing with different snapshot counts is crucial to assess the model’s performance under varying data availability, from limited snapshots (e.g., T=5) to more extensive datasets (e.g., T=1000), simulating real-world conditions and challenges.

T3. Various SNRs

This test set consists of samples generated by simulating signals with SNR levels different from those used in the training set, specifically {−20,−15,−5,5,15,20} dB. Testing with unseen SNR levels is crucial to evaluate the model’s generalization ability in real-world scenarios, where signal quality can vary significantly. Low SNR levels (e.g., −20dB) represent highly noisy environments, challenging the model’s robustness and noise tolerance, while higher SNR levels (e.g., 20dB) simulate clearer signals where precision becomes key.

T4. Off-grid angles

This test set comprises samples with arrival directions that are not part of the training grid (off-grid angles), specifically $\left[-59.7^{\circ },+58.3^{\circ }\right]$. Experimenting with off-grid signals is essential for evaluating the model’s ability to generalize beyond predefined scenarios and handle real-world conditions where signal arrival angles rarely align perfectly with the training grid.

T5. Combination of T2, T3 and T4

This test set combines the characteristics of the T2, T3, and T4 test sets, meaning all challenging conditions are present simultaneously. This makes the evaluation data highly realistic, as they reflect real-world scenarios where such conditions often co-occur.

T6. Various phases

This test set consists of signals with phase shifts in the range $\left[45^{\circ },90^{\circ },180^{\circ }\right]$, which are not present in the training set or the T1 test, where the phase is fixed at $0^{\circ }$. Testing with varying phase values is essential to assess the model’s robustness to phase variations, a common occurrence in real-world communication and radar systems due to factors like signal propagation and modulation schemes.

T7. Various frequencies

This test set is designed to evaluate the robustness of the models with respect to changes in signal frequency. The same parameters as in T1 were used, except that the frequency varies. Different carrier frequencies were applied while keeping the sensor positions fixed. Each frequency has its own wavelength and wavenumber, meaning the inter-element spacing is not normalized to each signal’s wavelength. Four subsets were created, each with signals at a different frequency: 2.4 GHz, 3 GHz, 5 GHz, and 10 GHz. These frequencies were selected due to their widespread use across various applications. Higher frequencies (5 GHz and 10 GHz) are typically better suited for short-range, high-bandwidth applications, while lower frequencies (2.4 GHz and 3 GHz) offer greater coverage and signal penetration.

T8. Modulation types

This test set is similar to T1, except that different modulation types are applied to simulate a variety of signal behaviors. Amplitude Modulation (AM) introduces a variation in the signal’s amplitude over time, creating fluctuations in strength that can be detected and analyzed. Frequency Modulation (FM) produces a signal with a varying instantaneous frequency, altering how rapidly the signal’s phase evolves, which affects its spectral properties. Phase Shift Keying (PSK) changes the signal’s phase according to a specific modulation scheme, typically used to encode information in discrete phase shifts, making it ideal for digital communication.

### 5.2. Results

The performance of the proposed model for DOA estimation is compared with five state-of-the-art DOA methods that have publicly available implementations. MUltiple SIgnificant Classification (MUSIC) identifies DOAs in correspondence with the pseudo-spectra identified on a predefined grid [5]. Root MUSIC (R-MUSIC) estimates the angular directions from the solutions of higher-order polynomials [61]. The aforementioned methods are covariance-based techniques that demand sufficient data snapshots for accurate DOA estimation and often assume known NOS. To ensure consistency with the experimental setup of other methods where the NOS is unknown, we applied the Akaike Information Criterion (AIC) to estimate the NOS before performing DOA estimation [62]. Multi-snapshot Newtonized Orthogonal Matching Pursuit (MNOMP) uses Newton refinement and feedback strategy for DOA estimation, leveraging Fast Fourier Transform (FFT) to keep computation complexity low [63]. The Multi-Task Autoencoder with Parallel multilayer Classifiers (MTAPC) model effectively estimates spatial spectra in complex scenarios, primarily thanks to the enhanced representation achieved by the multi-task autoencoder [9]. DNNDOA [18] is a CNN designed for DOA estimation in the low-SNR regime as a multi-label classification task, considering an on-grid approach.

The Cramér–Rao Lower Bound (CRLB) provides a theoretical benchmark for the minimum variance of unbiased estimators, making it an essential measure for complementing and validating the results obtained by existing methods in DOA estimation. Comparing DOA methods with the CRLB helps assess their proximity to optimal performance, highlighting both efficiency and unbiasedness. Since the CRLB accounts for factors such as noise and sensor configuration, it serves as a crucial reference for validating results on simulated data under various conditions. Methods that approach the CRLB reach the theoretical performance limit, while those that exceed it reveal areas for improvement [64]. Therefore, we include the CRLB in our comparison of results.

The results are presented using RMSE to evaluate the precision of DOA estimation, while accuracy is employed to estimate the NOS, measured as the fraction of correctly estimated sources. RMSE is calculated using the following equation [26]:

(11) $$\mathrm{RMSE}=\sqrt{\frac{1}{K}\sum_{k=1}^{K}\min_{j}{\left(d_{k}-{\hat{d}}_{j}\right)}^{2}},$$

where $d_{k}$ indicates the predicted angle, ${\hat{d}}_{j}$ represents the ground-truth angle, and *K* is the number of signals. Ground-truth and predicted signals are paired using a nearest-neighbor metric, ensuring that each predicted angle is compared to its closest ground-truth angle, resulting in a more accurate and lower RMSE value. When the number of predicted sources does not match the number of ground-truth sources, special handling is required for RMSE computation. Table 2 provides an example to demonstrate how these cases are addressed.

**Figure 3:**
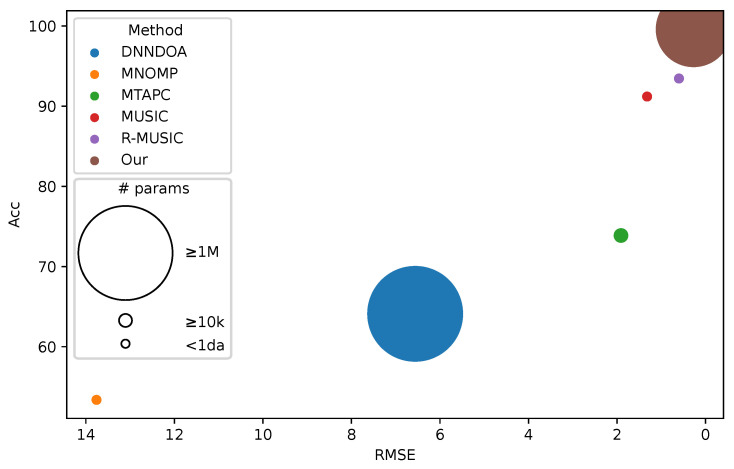
Ball chart reporting the RMSE versus accuracy. The size of each ball corresponds to the number of model parameters.

**Figure 4:**
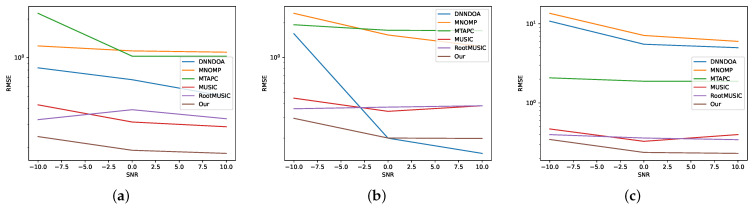
The DOA estimation performance for the T1 test set at varying SNRs divided by (**a**) one signal only, (**b**) two signals, and (**c**) three signals.

For test set T1, we report results in Table 3. As it is possible to see, the proposed method achieved the best performance with respect to the competitors. On the other hand, MNOMP achieves the worst RMSE and accuracy. From the perspective of efficiency, we evaluate methods based on inference time, computational complexity, and the number of parameters. In terms of inference time, deep learning methods are noticeably slower than traditional approaches like MUSIC, R-MUSIC, and MNOMP. When measuring computational complexity in Mega-FLOPS (MFLOPS), these traditional methods again prove more efficient than deep learning approaches. Among the deep learning methods, DNNDOA has the highest computational complexity, reaching 2.43 MFLOPS. Despite the fact that our proposed method has significantly more parameters than MUSIC, R-MUSIC, and MTAPC, Figure 3 demonstrates its superior performance in both RMSE and accuracy. Interestingly, DNNDOA, which has a similar parameter count to our model, performs the worst in terms of accuracy. In Figure 4, the RMSE is decomposed by the number of sources and varying SNR. Notably, the RMSE value for three sources, shown in panel (c), is higher than that for one and two sources. Among the algorithms evaluated, DNNDOA exhibits the greatest sensitivity to high SNR values.

In the T2 test set (see Table 4), performance declines with shorter snapshots for all methods. Our method outperforms others, with RMSE and accuracy of 11.81 and 47.04 (T=5) compared to 0.32 and 99.58 (T=1000). In the T3 test set, as seen in Table 5, the methods exhibit comparable RMSE performance, with the exception of DNNDOA. MTPAC achieves the lowest error, followed by R-MUSIC and MUSIC, with our method close behind. This suggests that the other methods demonstrate slightly better generalization to noise levels not covered in the training set.

The performance of the T4 test set is presented in Table 5. As before, it clearly demonstrates that the proposed method outperforms the other methods. In a broader context, when comparing these results with those in Table 3, it turns out that off-grid angles have a minimal impact on the performance of the methods under consideration. Analyzing the results presented in Table 6 for the T5 test set, it is evident that the proposed method’s RMSE performance remains relatively stable across varying snapshot lengths. In fact, our method outperforms the competitors in most cases, with the exception of RMSE at T=1000. It is worth noting the stark underperformance in estimating the NOS by the MUSIC and R-MUSIC methods when dealing with snapshots of fewer than 500, with rates as low as 0.00%. In Table 7, it achieves the lowest RMSE and high accuracy across all phases, proving robust against phase variability, while other methods like DNNDOA and MNOMP display variable performance with higher RMSE, particularly at 45° and 90° phases. In Table 8, the proposed method excels across the frequency spectrum, especially at higher frequencies (5 GHz and 10 GHz), where it achieves an RMSE of 18.56 and nearly 93% accuracy. While MUSIC and R-MUSIC maintain high accuracy, they show slightly higher RMSE, indicating some limitations at high frequencies. Table 9 shows that the proposed method also leads in handling different modulation types, particularly FM and PSK, achieving low RMSE and high accuracy.

**Figure 5:**
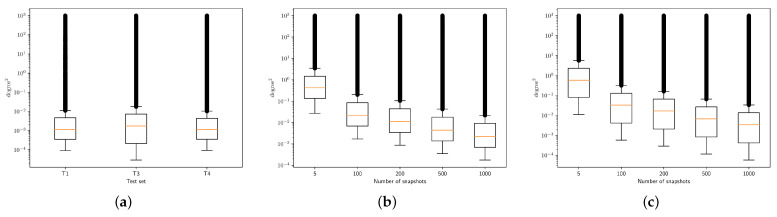
Boxplots showing CRLB index distributions for (**a**) test sets T1, T3, and T4, (**b**) various snapshots within T2, and (**c**) various snapshots within T5.

The boxplots in Figure 5 illustrate the CRLB index distributions for the different test sets and snapshot counts. In the first plot (T1, T3, T4), the CRLB values are concentrated between $10^{-3}$ and $10^{1}$ degrees squared. T3 shows a larger spread, indicating more variability in estimation error. In the second plot (T2), as the number of snapshots increases, the CRLB decreases significantly, reflecting improved estimation accuracy with more data. At 1000 snapshots, the CRLB is concentrated between $10^{-2}$ and $10^{-3}$. The third plot (T5) follows a similar trend, though with higher overall CRLB values compared to T2, indicating more challenging estimation conditions for T5. Overall, more snapshots lead to a lower and narrower CRLB, highlighting improved performance.

By comparing CRLB values with those achieved by the proposed method on the various test sets, it is possible to notice that as snapshot count increases, the RMSE of the proposed DOA estimation method converges toward the CRLB, indicating more efficient performance with larger data samples. At low snapshot counts (5 and 100), the RMSE is notably higher than the CRLB, reflecting increased estimation uncertainty with limited data. However, with more snapshots (200, 500, and especially 1000), the RMSE closely approaches the CRLB, demonstrating the method’s ability to achieve near-optimal accuracy when sufficient data are available. For instance, with 1000 snapshots in T2, the RMSE of 0.32 is close to the CRLB of 0.22, and in T5, the RMSE of 5.31 is near the CRLB of 5.72.

**Figure 6:**
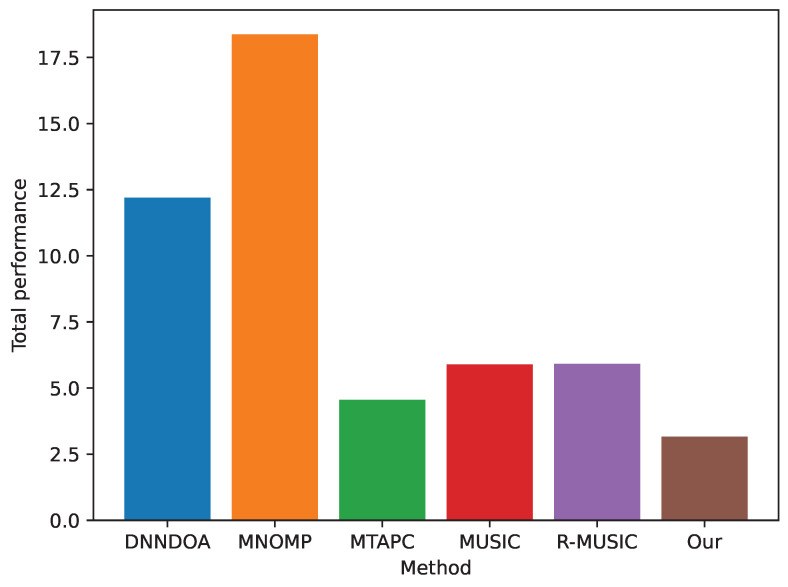
Scenario-independent total performance comparison.

To obtain a scenario-independent comparison of each method’s relative performance, we normalize the RMSE of each method by the CRLB for the corresponding scenario. Since the CRLB represents the best possible performance achievable in a given scenario, dividing the squared error by this bound yields a normalized measure, which is independent of the specific scenario’s characteristics. This normalization minimizes the impact of scenario variability on the RMSE, thus reducing the overall uncertainty when averaging performance across scenarios. The result is an effectiveness metric for each method, defined as the ratio of RMSE to CRLB, averaged over all scenarios. As seen in Figure 6, our approach more accurately reflects the performance disparities among estimators, making it evident how each one approaches or deviates from the theoretical CRLB limit.

### 5.3. Ablation Studies

In this section, we present the results of two ablation studies to evaluate the impact of training the proposed method in stages and varying the weights of the loss functions. In the first experiment, the learning process is divided into two steps. First, only the backbone and NOS branch are trained. Then, in the second step, the DOA branch is trained, leveraging the weights previously learned by the backbone and NOS estimator, without fine-tuning them. This incremental training approach allows us to evaluate the potential disadvantages of two-step training for DOA estimation compared to multi-task training, where shared features are learned simultaneously. In the second experiment, we explore various configurations of the $\lambda_{1}$ and $\lambda_{2}$ weights in Equation (8) to assess their impact on optimization and accuracy. To this end, we present results for two configurations that significantly differ from the optimal one: $\lambda_{1}=0.2$, $\lambda_{2}=1.0$, and $\lambda_{1}=1.0$, $\lambda_{2}=0.4$.

Results from the T1 test set, i.e., data with the same characteristics as the training set, are reported in Table 10. As it is possible to see, the two-step training approach results in a significantly higher RMSE (32.99) compared to the multi-task approaches, suggesting that learning the shared features simultaneously, as in multi-task training, leads to better performance for DOA estimation. The two configurations with different $\lambda_{1}$ and $\lambda_{2}$ weights show substantial differences in RMSE values. The configuration with $\lambda_{1}=0.2$ and $\lambda_{2}=1.0$ results in a higher RMSE (0.75) compared to the configuration with $\lambda_{1}=1.0$ and $\lambda_{2}=0.4$ (0.68), indicating that adjusting the balance of loss functions can improve optimization and reduce error. The proposed method (“Our” model version) achieves the best performance, with the lowest RMSE (0.27) and the highest accuracy (99.58%), suggesting that the specific configuration used in the final model optimally balances the training dynamics and yields the most accurate predictions.

## 6. Conclusions

In this paper, we proposed a deep multi-task CNN for joint NOS and DOA estimation. Compared with methods in the literature, the proposed method considers the estimation of the NOS as ordinal regression and the estimation of DOA as regression. The previous choices demonstrate the effectiveness of the proposed method even under challenging conditions. As future work, we aim to improve the proposed method by incorporating additional signal properties, such as noise statistics, signal coherence, and temporal snapshot information, to enhance the model’s ability to capture complex data relationships and improve DOA estimation accuracy in challenging environments, as seen in classical detection theory methods. While our current tests include varying SNRs, off-grid angles, and snapshots, future experiments will also involve environmental factors such as multipath propagation, receiver sampling ratio, and ADC quantization bits. Additionally, we plan to validate the method using real-world data, where obstacles and reflections are harder to simulate but crucial for practical applications. This will help refine our approach for real-world scenarios and ensure robustness in diverse environments.

## Figures and Tables

**Table 1:** The backbone architecture proposed in this work. Each row describes an operation by specifying the input shape, operator type, number of output channels (c), stride (s), and output shape. A dash “-” indicates that the operator does not require a value for the parameter.

Input	Operator	c	s	Output
2×M×M	Conv2d	128	2	128×M/2×M/2
128×M/2×M/2	BatchNorm2d	-	-	128×M/2×M/2
128×M/2×M/2	ReLU	-	-	128×M/2×M/2
128×M/2×M/2	Conv2d	64	1	64×M/2×M/2
64×M/2×M/2	BatchNorm2d	-	-	64×M/2×M/2
64×M/2×M/2	ReLU	-	-	64×M/2×M/2
64×M/2×M/2	Conv2d	64	1	64×M/2×M/2
64×M/2×M/2	BatchNorm2d	-	-	64×M/2×M/2
64×M/2×M/2	ReLU	-	-	64×M/2×M/2
64×M/2×M/2	Flatten	-	-	64∗M/2∗M/2

**Table 2:** Samples illustrating how RMSE is computed when the number of predicted sources differs from the number of ground-truth sources. In these examples, $\hat{d}$ represents the ground-truth DOA, and *d* represents the predicted DOA.

Case	Direction	RMSE
$|\hat{d}\left|\;<\;|d\right|$	$\hat{d}=\left\{15,-19\right\}$ d={−20,15,32}	$\sqrt{\frac{{\left(15-15\right)}^{2}+{\left(-19+20\right)}^{2}}{2}}=0.71$
$|\hat{d}\left|\;>\;|d\right|$	$\hat{d}=\left\{-19,42,15\right\}$ d={−20,15}	$\sqrt{\frac{{\left(-19+20\right)}^{2}+{\left(42-15\right)}^{2}+{\left(15-15\right)}^{2}}{3}}=15.60$

**Table 3:** Results reported in terms of RMSE, accuracy (%), inference time (ms) and computational complexity (MFlops) for the test set with the same configuration as the training set. The best result for each metric is shown in **boldface**.

Method	RMSE (↓)	Accuracy (↑)	Inference Time (↓)	Computational Complexity (↓)
DNNDOA [18]	6.56 ± 15.55	64.10	40.46	2.43
MNOMP [63]	13.76 ± 18.39	53.37	1.92	**0.01**
MTAPC [9]	1.91 ± 7.36	73.87	40.27	1.61
MUSIC [5]	1.32 ± 2.56	91.20	1.45	0.02
R-MUSIC [61]	0.60 ± 1.37	93.45	**0.74**	0.02
Our	**0.27**± 0.21	**99.58**	40.22	1.11

**Table 4:** RMSE and accuracy for different *T* snapshots. The best result for each metric is shown in **boldface**.

Method	5		100		200		500		1000	
	RMSE (↓)	Acc. (↑)	RMSE (↓)	Acc. (↑)	RMSE (↓)	Acc. (↑)	RMSE (↓)	Acc. (↑)	RMSE (↓)	Acc. (↑)
DNNDOA [18]	30.71 ± 17.32	6.41	14.65 ± 19.53	40.64	11.17 ± 18.28	49.60	8.07 ± 16.61	58.30	7.05 ± 15.92	61.87
MNOMP [63]	48.56 ± 25.25	6.76	38.05 ± 24.54	42.27	36.07 ± 24.30	55.40	35.92 ± 23.93	59.70	31.73 ± 22.18	66.31
MTAPC [9]	16.19 ± 10.02	32.51	2.40 ± 7.49	63.23	2.23 ± 7.53	68.76	2.07 ± 7.49	72.45	1.98 ± 7.42	73.44
MUSIC [5]	19.42 ± 20.85	2.01	3.05 ± 1.27	0.00	1.73 ± 0.96	0.01	0.61 ± 1.74	11.75	1.30 ± 1.66	85.34
R-MUSIC [61]	19.72 ± 21.29	1.03	3.12 ± 2.10	0.00	1.79 ± 0.86	0.01	0.56 ± 0.51	11.75	1.25 ± 1.42	85.45
Our	**11.81** ± **9.59**	**47.04**	**2.24** ± **3.29**	**91.46**	**1.13** ± **0.86**	**97.57**	**0.47** ± **0.45**	**99.46**	**0.32** ± **0.31**	**99.58**

**Table 5:** RMSE and accuracy for various SNRs (i.e., −20, −15, −5, 5, 15, 20) and off-grid angles (i.e., between $-59.7^{\circ }$ and $+58.3^{\circ }$). The best result for each metric is shown in **boldface**.

Method	Various SNRs		Off-Grid Angles	
	RMSE (↓)	Accuracy (↑)	RMSE (↓)	Accuracy (↑)
DNNDOA [18]	29.82 ± 15.46	4.52	8.31 ± 16.90	51.17
MNOMP [63]	34.69 ± 31.16	12.62	9.98 ± 7.51	56.20
MTAPC [9]	**2.24** ± **7.18**	61.52	1.72 ± 6.39	73.66
MUSIC [5]	6.02 ± 14.55	74.67	0.34 ± 0.68	90.81
R-MUSIC [61]	6.02 ± 14.62	74.73	0.32 ± 0.39	92.34
Our	8.47 ± 14.04	**87.77**	**0.26** ± **0.23**	**99.57**

**Table 6:** RMSE and accuracy for the test set with different *T* snapshots, off-grid angles and various SNRs. The best result for each metric is shown in **boldface**.

Method	5		100		200		500		1000	
	RMSE (↓)	Acc. (↑)	RMSE (↓)	Acc. (↑)	RMSE (↓)	Acc. (↑)	RMSE (↓)	Acc. (↑)	RMSE (↓)	Acc. (↑)
DNNDOA [18]	32.60 ± 14.31	1.94	29.91 ± 15.46	4.35	29.68 ± 15.31	4.46	29.60 ± 15.27	4.47	29.59 ± 15.26	4.46
MNOMP [63]	46.28 ± 25.24	9.02	32.54 ± 23.70	37.07	30.84 ± 23.33	44.93	30.18 ± 23.28	48.45	28.38 ± 16.75	54.19
MTAPC [9]	17.16 ± 12.53	31.82	13.61 ± 11.70	50.60	13.08 ± 11.15	51.90	12.54 ± 10.61	54.82	12.23 ± 10.37	57.95
MUSIC [5]	20.34 ± 21.47	1.03	18.83 ± 18.44	0.00	18.48 ± 16.17	0.00	20.02 ± 15.98	14.93	15.76 ± 14.11	70.26
R-MUSIC [61]	20.91 ± 18.39	0.02	18.71 ± 16.02	0.00	18.44 ± 16.95	0.00	20.31 ± 15.84	14.69	15.81 ± 14.43	70.32
Our	**12.20** ± **9.78**	**44.68**	**8.74** ± **13.90**	**65.78**	**8.39** ± **13.23**	**66.23**	**7.90** ± **12.73**	**76.69**	**6.31** ± **11.61**	**85.44**

**Table 7:** RMSE and accuracy for various phases of test set T6. The best result for each metric is shown in **boldface**.

Method	0		45		90		180	
	RMSE (↓)	Acc. (↑)	RMSE (↓)	Acc. (↑)	RMSE (↓)	Acc. (↑)	RMSE (↓)	Acc. (↑)
DNNDOA [18]	6.56 ± 15.55	64.10	27.78 ± 17.86	46.25	32.79 ± 17.87	49.29	22.77 ± 15.82	42.36
MNOMP [63]	13.76 ± 18.39	53.37	62.67 ± 20.12	34.67	58.91 ± 18.29	32.02	71.01 ± 18.09	38.02
MTAPC [9]	1.91 ± 7.36	73.87	22.97 ± 15.29	56.59	29.10 ± 12.29	51.42	18.91 ± 11.01	58.12
MUSIC [5]	1.32 ± 2.56	91.20	21.87 ± 15.20	91.71	24.09 ± 12.09	92.01	**18.83** ± **12.51**	90.98
R-MUSIC [61]	0.60 ± 1.37	93.45	27.38 ± 11.05	91.71	29.12 ± 11.42	92.61	34.10 ± 15.21	89.08
Our	**0.27** ± **0.21**	99.58	**7.12** ± **9.12**	92.18	**15.91** ± **14.61**	92.61	19.06 ± 13.24	90.49

**Table 8:** RMSE and accuracy for various frequencies. The best result for each metric is shown in **boldface**.

Method	1 GHz		2.4 GHz		3 GHz		5 GHz		10 GHz	
	RMSE (↓)	Acc. (↑)	RMSE (↓)	Acc. (↑)	RMSE (↓)	Acc. (↑)	RMSE (↓)	Acc. (↑)	RMSE (↓)	Acc. (↑)
DNNDOA [18]	6.56 ± 15.55	64.10	28.57 ± 16.89	47.01	27.39 ± 17.69	45.28	26.97 ± 18.02	47.86	27.20 ± 17.83	45.35
MNOMP [63]	13.76 ± 18.39	53.37	34.91 ± 21.21	49.12	34.20 ± 18.91	51.21	32.00 ± 23.11	48.03	31.93 ± 21.43	39.55
MTAPC [9]	1.91 ± 7.36	73.87	**21.12** ± **15.22**	52.86	**19.78** ± **13.17**	52.57	20.55 ± 13.00	52.00	20.44 ± 13.47	49.88
MUSIC [5]	1.32 ± 2.56	91.20	22.76 ± 14.64	92.88	23.11 ± 16.37	91.56	24.52 ± 16.45	90.63	24.80 ± 17.13	91.38
R-MUSIC [61]	0.60 ± 1.37	93.45	31.43 ± 11.56	92.22	30.92 ± 9.76	91.56	31.81 ± 9.64	90.63	32.53 ± 9.63	91.52
Our	**0.27** ± **0.21**	99.58	24.52 ± 13.78	92.45	21.46 ± 12.80	93.01	**19.91** ± **12.46**	94.63	**18.56** ± **10.82**	92.74

**Table 9:** RMSE and accuracy for three modulation types in test set T8. The best result for each metric is shown in **boldface**.

Method	AM		FM		PSK	
	RMSE (↓)	Acc. (↑)	RMSE (↓)	Acc. (↑)	RMSE (↓)	Acc. (↑)
DNNDOA [18]	22.22 ± 12.83	48.38	26.02 ± 17.82	45.41	49.11 ± 21.82	43.34
MNOMP [63]	36.82 ± 23.60	44.12	31.69 ± 29.14	47.23	51.11 ± 11.29	49.92
MTAPC [9]	19.43 ± 8.48	62.88	22.91 ± 10.17	49.89	29.08 ± 15.22	53.24
MUSIC [5]	18.42 ± 11.15	91.37	22.84 ± 13.02	93.92	29.83 ± 17.12	91.38
R-MUSIC [61]	26.53 ± 9.63	91.37	27.91 ± 11.01	92.82	32.53 ± 9.63	91.38
Our	**16.57** ± **11.76**	92.74	**19.82** ± **10.05**	92.22	**23.57** ± **12.97**	91.43

**Table 10:** Ablation results. The best result for each metric is shown in **boldface**.

Model Version	RMSE	Accuracy
Two-step training	32.99 ± 9.61	98.86
Loss function with $\lambda_{1}=0.2$ and $\lambda_{2}=1.0$	0.75 ± 0.40	98.64
Loss function with $\lambda_{1}=1.0$ and $\lambda_{2}=0.4$	0.68 ± 0.38	99.13
Our	**0.27** ± **0.21**	**99.58**

## Data Availability

Data are contained within the article.
